# Investigating inequalities in admissions of young people out-of-area or to adult psychiatric wards: a qualitative study

**DOI:** 10.1007/s00787-025-02849-9

**Published:** 2025-09-09

**Authors:** Leonie Lewis, Josephine Holland, James Roe, Camilla Parker, Kapil Sayal

**Affiliations:** 1 Mental Health and Clinical Neuroscience, Institute of Mental Health, School of Medicine, University of Nottingham, Nottingham, UK; 2 National Institute for Health and Care Research, Applied Research Collaboration East Midlands, University of Nottingham, Nottingham, UK; 3 Just Equality: Research, Training and Consultancy, Norwich, England, UK

**Keywords:** Inpatient care, Out-of-area admissions, Carers, Looked-after children, Inequalities

## Abstract

**Background:**

High occupancy rates and uneven bed distribution throughout England mean some young people are admitted to Child and Adolescent Mental Health Service beds out-of-area or to adult psychiatric wards in Adult Mental Health Services. No previous research has investigated how the characteristics of young people, families, or services may influence the impact of these types of admissions.

**Method:**

Data were collected as part of the “Far Away from Home” research program. Semi-structured interviews were carried out with 13–17-year-olds (*n* = 30), parents/carers (*n* = 21) and healthcare professionals (*n* = 68) who had experience of out-of-area, local or adult psychiatric ward admissions. Thematic analysis of transcripts identified characteristics which positively or negatively moderated the impact of these admissions.

**Results:**

Young people in the care of the state (looked-after children) and those from more disadvantaged families with fewer financial resources, low social capital, health problems and disability were found to be more negatively affected by out-of-area admissions. In adult ward admissions, less mature young people were found to be more negatively affected. Service characteristics which lessened these negative effects were identified, such as: flexible visiting times, transport, financial reimbursement or accommodation for visiting parents. For families with complex dynamics being admitted locally was sometimes felt to be negative.

**Conclusions:**

The impacts of admissions to out-of-area or adult wards are not equal in all circumstances. Potential inequalities should be considered before and during admission and throughout discharge processes, with steps taken to support disadvantaged groups.

## Introduction

Increasing numbers of children and young people (YP) are developing mental health disorders, requiring referral to child and adolescent mental health services (CAMHS) [1, 2]. This is placing pressure on both community and inpatient services [3] as well as others such as acute hospital wards [4] and the police [5].

Steps have been taken to reduce the geographical variation in adolescent inpatient bed availability across England [6]. However, out-of-area (more than 50 miles from home address or out-of-region [7]) and adult mental health unit admissions continue to be required to meet the demand for inpatient beds [8]. Out-of-area admissions to psychiatric units for any age group have been labelled as “inappropriate” since they separate an individual, who is already struggling, from their support networks [9, 10]. Admissions of under-18s to adult psychiatric wards has been similarly labelled “inappropriate” due to concerns about young people being exposed to very unwell adults and difficulties in providing access to education and age-appropriate activities [11]. NHS best practice guidance emphasises the importance of minimising out-of-area placements and prioritising local, community-based care [12]. These sentiments have also been echoed by the Children’s commissioner and the Care Quality Commission (CQC) who raise concerns about admissions both out-of-area and to adult wards [13, 14]. The NHS Long Term Plan aimed to eliminate out-of-area placements for adults and children by 2020/21 but this target has not been met [15]. In England, mental health legislation (section 131 A of the Mental Health Act 1983) provides that all patients aged under-18 should be treated in an environment in hospital which is suitable for their age [16].

The ‘Far Away from Home’ study is a multi-methods programme of research investigating the impacts of admission of under-18s to CAMHS inpatient wards out-of-area or adult mental health wards. Within one part of this study, data about the demographics, clinical characteristics and 6-month follow-up outcomes for 290 out-of-area or adult ward admissions were collected [8]. Findings showed that on average admissions out-of-area were 14 days longer, with delays to discharge in over a third of admissions [8]. The primary qualitative paper identified that out-of-area admissions may: exacerbate distress and uncertainty, limit support networks, cause practical challenges for families visiting, and create difficulty for clinicians to ensure continuity of care [17]. A companion paper looking at young people admitted to adult wards raised concerns about young people’s safeguarding, access to education, and lack of treatment [18].

Although out-of-area and adult ward admissions in CAMHS have been labelled as inappropriate, the impacts of such admissions are likely to be affected by characteristics of the young person, their family, and the network of services around them. Inequalities in mental health care experiences are well documented [19]. However, no previous research has investigated what characteristics are associated with inequalities in mental healthcare experiences during these types of admissions. This study aims to identify the young person, family and service characteristics that affect the impact of an out-of-area or adult ward admission, either positively or negatively. It also aims to identify ways in which services may mitigate some of these inequalities that impact these admissions.

## Methods

### Study design and setting 

This work forms part of the ‘Far Away from Home’ study [8], a multi-methods programme of research investigating the impacts of admissions young people to out-of-area CAMHS inpatient wards and adult psychiatric wards. The findings from associated studies are reported elsewhere [8, 17, 18].

### Ethical approval and informed consent statements

The authors assert that all procedures contributing to this work comply with the ethical standards of the relevant national and institutional committees on human experimentation and with the Helsinki Declaration of 1975, as revised in 2008. All procedures involving human subjects/patients were approved by West Midlands—South Birmingham Research Ethics Committee 20/WM/0314. Participants aged 16 years and over gave written informed consent. Participants aged 13–15 years gave written informed assent combined with written informed consent from a parent/carer.

### Participants

Young people (aged 13–17 years), or the parent/carer of a young person, with a current or recent (within last 12 months) admission, were recruited. Some young people had been admitted to a General Adolescent Unit (GAU, a mental health unit for young people), that was either out-of-area (more than 50 miles from their home address or out-of-region) or to a unit in their local area. Other young people had been admitted to an adult mental health ward. Healthcare professionals (HCPs) were those involved with the admission, management, or care of the young person who had been admitted to an out-of-area general adolescent unit or to an adult ward.

Young people and parents/carers were recruited from mental health inpatient and community settings in five large regions of England (East Midlands, West Midlands, East of England, Greater Manchester, and Oxford and Thames Valley). HCPs were recruited nationally and were identified through a national surveillance study [8], and by a snowball approach through local participating sites. Perspectives were sought from a range of HCPs including inpatient and community (private and NHS) consultant child and adolescent psychiatrists, general adult psychiatrists, nurses, ward managers, and service commissioners.

### Procedures

Interviews were conducted remotely over video call or telephone, or face-to-face in CAMHS settings. Topic guides were developed through collaboration with three study advisory groups, comprising of young people, parents/carers, and HCPs [17]. These guides aimed to explore experiences around admission, and perceptions on challenges, benefits and harms, and suggestions for improvement of services. At the start of each interview with a HCP, they were asked to recount an experience of looking after a young person admitted out-of-area or to an adult mental health ward.

Interviews were conducted by seven researchers (six female, one male) between March 2021 and September 2022. Each interview lasted between 25 and 60 min. Young people and parents/carers were given an online voucher (£15) in appreciation for their time.

### Data analysis

Interviews were audio recorded and transcribed verbatim. All transcripts were coded in NVivo V.12. Two researchers (LL and JH) independently coded the data. Two researchers (LL and JH) independently coded an additional 10 transcripts each (10%) for cross-checking.

The analysis of the qualitative data followed an inductive approach. Following a thematic approach [20] any data which referred to characteristics of a young person, their family or services which increased or decreased the impact of the admission being out-of-area or to an adult ward were coded. These preliminary codes were then grouped by the researchers (JH, LL, and JR) until saturation of the themes was found.

## Results

In total, 119 participants took part. Participant characteristics are detailed in Table 1.

**Table 1:** Participant characteristics

	Young person (YP) (*n* = 30)	Parent/carer (P/C) (*n* = 21)
Gender	Female = 22	Female = 16
Age: mean years; range	Mean = 15.9 Range = 13–17	Mean = 49.65 Range = 37–63
Ethnicity	White: English, Welsh, Scottish, Northern Irish or British = 19	White: English, Welsh, Scottish, Northern Irish or British = 12
Admission type	Out-of-area = 19 Near home = 7 Adult ward = 2 Adult & out-of-area = 2	Out-of-area = 9 Near home = 8 Adult ward = 2 Adult & out-of-area = 2
	Healthcare professional (*n* = 68)	
Role	Community consultant in child and adolescent psychiatry = 19 Inpatient consultant in child and adolescent psychiatry = 17 Inpatient consultant in adult psychiatry = 10 Ward/case manager = 7 Mental health commissioner = 4 Nurse in adult mental health = 4 Clinical nurse lead/senior nurse/specialist nurse = 4 Private sector consultant in child and adolescent psychiatry = 3	

### Summary of themes identified

Four key themes were identified which increased the negative impact of being admitted out-of-area or to an adult ward. The identified themes included: (1) being under the care of the state, (2) being from a family with limited resources, (3) having significant relational issues and (4) being younger or more immature. When considering what characteristics decreased the impact of being admitted out-of-area or to an adult ward, all data coded described ways in which services could improve experiences. These were therefore grouped under one theme of service factors that helped to mitigate the impact of out-of-area or adult ward admission. Data were also coded for mentions of characteristics which affected the impact of near home admission.

### Characteristics which increased the negative impact of being admitted out-of-area or to an adult ward

#### Being under the care of the state (Looked-after children)

When asked to describe a case where a young person had been admitted out-of-area or to an adult ward, healthcare professionals frequently chose cases involving looked-after children. Through their descriptions of these cases, they highlighted a number of characteristics which are unique or common within this population, which amplified the negative impacts of the admission being out-of-area or to an adult ward. These findings were also reflected in the data collected from young people, some of whom were looked-after.

On a practical level participants noted that due to the limited number of care placements and the risk of placement breakdown, looked-after children may already be living at considerable distance from home at the time of their admission.

*“I mean I think the looked-after population often disproportionately crop up in the out-of-area ones because they’re placed in a placement somewhere not near home.”* (HCP).

*“…basically I was in so many different placements….I was in [Name of town] in a residential care home when I was in [local Adult Ward] but then they got rid of me. Then when I was in [Name of another Adult ward] my placement was in [other UK country].”* (YP).

Services could therefore sometimes struggle to identify what should be counted as the local general adolescent unit, whether it was close to the current residence of the young person or the residence of the family of origin. Since looked-after children have been removed from their family of origin, their familial relationships were particularly complex. The strained nature of this relationship often interacted with the distance to reduce the amount of visits a young person had.

*“She was a looked-after child…. family did no visits whatsoever at all during her entire stay because of the duration of time that they had to put in”* (HCP).

Looked-after children who are admitted to a mental health unit have frequently experienced developmental trauma within their family of origin and breakdown of care placements. As a result of these experiences, these young people were often perceived by staff to be less emotionally mature than their same-aged peers.

*“Developmentally they’re actually probably quite a bit younger because their childhood’s been disrupted and disturbed and maybe they’ve had experience of trauma.”* (HCP)

*“I’m in the care system*,* so when I do make relationships with people and I trust people*,* they just walk out of my life. So it’s really hard for me to actually build trust up with people because they just walk out.”* (YP).

Some healthcare professionals reflected how uniquely frightening it was for these young people during these admissions to have no trusted adult to call.

*“How frightening is that situation for a 14-year-old with no family to call?”* (HCP talking about a looked-after young person admitted to adult ward).

#### Being from a family with limited resources

Having your child admitted out-of-area increased difficulties for family members making visits and facilitating leave. All groups interviewed described how the impact of this was greater for families with fewer resources. Parents described the financial burden of travelling, which disproportionally impacted low-income families.

*“Not all these parents have the finances to be able to drive up and down the motorway. There is no support financially for parents to do that… I’ve only known a couple of occasions where Social Care have been able to give parents like bus money and stuff like that.” (HCP)*.

Where parents could not drive or afford a car, travelling times and journey costs were often increased due to the need to use public transport.

*“My parents don’t drive*,* so to get to a hospital in [Name of city] two hours away we didn’t really have any way to get there.”* (YP).

*“Her mum takes all day to get here on a coach*,* for a couple of hours’ visit*,* to go all the way back home. That can only happen twice a week”* (HCP).

This also affected whether parents were able to facilitate leave. This often led to young people of low-income families being unable to have as many trials of leave as peers who were admitted locally or who had more affluent parents. This placed an additional pressure on clinicians when making decisions around discharge and whether it was safe.

*“They wanted us to go down every weekend or….they probably thought I was really uncaring because I couldn’t do it. I just couldn’t afford to do it…”* (P/C).

*“A risk-benefit kind of assessment in terms of you know*,* to put that girl on a coach with her mum down a motorway….I think it adds really quite a harmful impact in terms of that.”* (HCP).

When there was an acute petrol shortage additional strain was placed on families.

*“There was this like petrol shortage and they couldn’t come”* (YP).

It was not only financial factors which determined how well-resourced a family was and how much they were affected. Parents were often working and caring for other children or relatives as well as trying to support their child in hospital. How supportive their workplace was or the presence of a local support network to help with their care needs determined how much they could visit or facilitate leave.

*“The other kids finish school at half 3*,* we had to get other people involved and help us a little bit picking the kids up and looking after the kids until we got back.” (P/C)*.

*“He was trying to hold down a job which he’d just got and you could see that he was so scared of losing his job through having to*,* you know*,* come out of work to attend meetings*,* to attend child protection meetings*,* all these…”* (HCP).

Young people described their worries about parents with disabilities or health problems being able to visit them.

*“Yeah*,* so she struggles to walk a lot. So having to travel that far she’s in a lot of pain during our visits*,* and she has to go back as well.”* (YP).

One HCP described a young person who was unable to visit their dying mother due to the distance from home:

*“The team is far away; it makes it really difficult and now this young person is in hospital not knowing that his mum is going to pass away. The family being unable to come here to take him home.” (HCP)*.

If a young person and their family were homeless, this further exacerbated the negative impact of the out-of-area admission because units struggled to form links with local social care teams. There was a report of a young person going on day leave to a hostel which was attributed to exacerbating his psychosis:

*“Within a month of being in that hostel…every time we sent him on day leave home*,* he would come back really ill and take about a week to recover from being at home for a few hours” (HCP)*.

In contrast, for young people admitted locally, even when parents were working, visits and leave were less impacted.

*“Leave is alright as well because it’s pretty easy for me to go in between mealtimes because I literally live down the road.”* (YP).

*“And it was easier for my parents to visit me when I first got here because it was a lot closer. And they can like visit me more often because my mum works*,* so she’s got to visit around work time as well.”* (YP).

#### Having significant relational issues (young person themselves or within the family)

As described in a previous paper [17], for some young people with complex family dynamics or safeguarding issues, admission locally could be felt to be more negative.

*“Families that are quite enmeshed and there’s quite high levels of anxiety*,* that can be quite difficult when they’re local.”* (HCP).

However, others disagreed with this perspective and described how these factors also increased the impacts of out-of-area admission. If the young person was returning to live with their family, the combination of the complex family dynamics and the distance could make the provision of family interventions difficult.

*“People talk about “parentectomies”*,* the idea that children need to be separated from their families in order to manage… children need to be protected from harmful people who could be on their doorstep but there are other ways of doing that without having to disconnect a child from where they live and everything about their community. And all those other safe adults who would be part of their network” (HCP)*.

Young people with a history of trauma, emotional dysregulation, attachment difficulties or emerging personality difficulties were identified as being difficult to find a bed for, and thus more likely to be placed further away. The combination of these difficulties and distance delayed discharges and increased lengths of admissions.

#### Being younger in age or more immature

Younger adolescents may have little or no experience of being away from their family for a prolonged period. The addition of a large physical distance to these early experiences increased parent/carer anxiety.

*“She was so scared when she first got sectioned… she’d never had any nights away let alone being taken into a locked-up hospital”* (P/C).

For those working on adult wards, staff described feeling unprepared to care for younger adolescents since they were different to their usual patients. Healthcare professionals described challenges in communication with younger people.

*“She was a very young 15*,* that I really felt out of my depth in just talking to her. Even though I have children of that age but obviously it’s a very different environment where we struggled to relate to her clinically.”* (HCP).

As noted in the above theme of children under the care of the state, according to the healthcare professional interviews, maturity was also a consideration, as well as chronological age, particularly for adult ward admissions.

*“…you can get a really immature 17-year-old that is very much a child and then you get some that are incredibly streetwise that have seen and done a lot more than I’d done at 21.”* (HCP).

### Characteristics which decreased the negative impact of being admitted out-of-area or to an adult ward

#### Service factors that helped to mitigate the impact of out-of-area or adult ward admission

As well as characteristics of the young people and families and how these affected the impact of the out-of-area or adult ward admission, within the data there were descriptions of how services could mitigate some of the negative impacts.

Where there was effective interagency working, the impact of the admission being out-of-area was decreased. Social care support was also a large factor in the success of admission. A consistent social worker or community worker who had good communication with the young person and HCPs was important. In contrast, a lack of availability or breakdown of social care placements was a barrier for discharge from out-of-area admission.

*“And I think one risk is that we sort of lose touch… it’s that out of sight out of mind thing , that’s what it feels like. And I think it’s easier for that to happen when people are far away” (HCP).*

*“…so my youth worker who’s pretty much my second mum because I’ve known her since I was 13. She comes up every week.” (YP).*

##### Phone use and video calls.

The ability for young people to stay in touch with their support network by phone was particularly important during out-of-area admissions. At times young people’s contact with family and friends were restricted due to ward rules, a lack of privacy or poor internet connection. Individuals also described mobile phone use being restricted as a sanction.

*“I found it very difficult when the young person was denied their mobile phone…When someone goes through mental health issues they say please don’t isolate*,* make sure you socialise*,* don’t lose connections and yet that was the punishment”* (P/C).

*“…it was literally like being in prison. Like I had no phone*,* wasn’t allowed a phone.”* (YP).

##### Reimbursement.

Parents and carers were very appreciative of services which could provide support with accommodation, transport or visiting expenses. HCP often described that there could be disagreements within the professional network about whether this reimbursement should be from health care or social care.

*“So sometimes your staff drive kids back to their parents’ house even if it’s sort of 60/70 miles? HCP: No*,* no*,* 200 miles*” (HCP).

### Characteristics which affected the impact of being admitted to a local ward

As noted above, for young people admitted to local wards, if there were safeguarding issues or complex family dynamics, sometimes this could add complexity.

However, no other specific characteristics of the young person, family or services were felt to affect the impact of the admission. Young people spoke positively of how proximity to home improved their access to visits, leave and re-integration into school.

*“…just because if it’s nearby it’s convenient. Especially for stuff like school visits because I think something that me and my family have had like sort of at the forefront of our minds is going back to school as soon as I could.”* (YP).

## Discussion

This study explored experiences, inequalities and negative impacts of out-of-area admissions and admissions to an adult ward. We found that these admissions are particularly negative for looked-after children, families with limited resources, young people and families with relational issues, and younger or more immature children. For young people with complex family dynamics or safeguarding concerns, being admitted locally was also felt to be negative. We identified several service characteristics which can help mitigate some of the impacts, including flexible visiting times, support for visiting parents, and permission to use phones and video calls to keep in contact.

Previous research has shown that looked-after children are more likely to experience educational, behavioural, physical, and psychological problems compared to peers [21]. They are also more likely to experience adverse adult socio-economic, educational, legal and health outcomes above those associated with co-existing childhood or adult disadvantage [22, 23]. The findings of this study further evidence how this particularly vulnerable group is disadvantaged when admitted to mental health wards and units. In recognition of the specific needs of this group, in some parts of the UK, specialist CAMHS teams for looked-after children have been commissioned. However, these findings suggest that all services need to be attuned to the needs of looked-after children, not only these specialist community teams.

The finding that these admissions have a higher impact on families with limited resources also aligns with the literature. As highlighted in the 2010 Marmot review [24, 25], and the Centre for Mental Health report on poverty and mental health [26], poverty, deprivation, and mental health are inter-linked. However, these findings also highlight that more deprived young people, if they are sent out-of-area or to an adult ward, are more likely to be negatively impacted. The Mental Health Act 1983 Code of Practice, paragraph 19.122 states that “local authorities should consider whether it would be appropriate to provide financial support to enable families to visit children and young people in hospital….The provision of financial support to cover the travel costs of visiting might be essential for some families on low incomes, especially if their child has been placed out of area” [16]. However, this wording promotes consideration only and does not mandate this provision, our data suggest that this funding is not being provided.

As noted within the interviews, removing a young person from their home places them in an artificial environment for their treatment. The young person and their family are then expected to form trusting, therapeutic relationships with many staff and peers. For young people and families who struggle with making and maintaining relationships this can be particularly difficult. When there is the added difficulty of these relationships being long distance, but temporary, it can further add to the strain. It can therefore be seen how there is negative interaction between the young person or their family having relational issues and the admission being out-of-area or to an adult ward.

For some younger adolescents, admission may be their first time away from home. It is understandable that being admitted a long distance away may increase the distress around this separation between the young person and their family. The interviewees also commented that it is not only chronological age which may determine how a child copes with being away from family but also their social and emotional maturity, the development of which may have been affected by developmental trauma or serious mental illness. This study did not capture what measures staff had put in place to be able to support these young people. It would be useful for future research to gather examples of what unit staff are able to do to specifically address this issue and offer additional support to younger or more immature young people.

Although the identified themes reflect primarily negative experiences and impact, the data also revealed ways in which services could help reduce the challenges of out-of-area admissions for young people and admissions to an adult ward. These include a focus on: effective communication, practical and financial support for parents, flexible visiting hours, education, access to mobile phones and video calls. According to the Mental Health Act Code of Practice all of these practices should be standard to every admission [16]. However, there is a greater need to implement these practices. This study has several strengths. It extends from previous research by not only considering the impacts of these admissions but also considering differential impacts on young people and families. The large sample size (*n* = 119) allowed us to explore rich and varied secondary data. The geographical national spread of participants, recruitment of participants from a range of social backgrounds, and HCPs from a variety of clinical roles and backgrounds allowed us to capture a diverse range of experiences. There are also limitations to be acknowledged. Through recruitment of young people from inpatient units, the majority of young people were still in hospital, potentially impacting their ability to reflect on discharge.

## Conclusion

In conclusion, this study has highlighted that although out-of-area and adult ward admissions have been deemed inappropriate for all young people, there are certain individuals and groups for whom the negative impact of the admission being out-out-of-area or to an adult ward is greater. Particular consideration should be paid, and policies may need to be developed to ensure that additional support is put in place to mitigate this health inequality. It has also highlighted that current practices frequently do not align with the Mental Health Act 1983: Code of Practice, particularly around support for the relationship between a young person and their family or carers whilst admitted. All services should be ensuring that they adopt the practices found to reduce the negative aspects of these admissions including ensuring access to video-calling and phones, and reimbursement of visiting expenses for parents on lower incomes.

## Data Availability

Data are available upon reasonable request from the corresponding author.
